# Laparoscopic Management of Cesarean Scar Pregnancy with Temporary Clipping of Anterior Trunk of Hypogastric Arteries: A Case Report

**DOI:** 10.3390/jpm14050469

**Published:** 2024-04-28

**Authors:** Ioana-Flavia Bacila, Ligia Balulescu, Alexandru Dabica, Simona Brasoveanu, Marilena Pirtea, Adrian Ratiu, Laurentiu Pirtea

**Affiliations:** Department of Obstetrics and Gynecology, Victor Babes University of Medicine and Pharmacy, 300041 Timisoara, Romania; ioana.bacila@umft.ro (I.-F.B.); alexandru.dabica@umft.ro (A.D.); simona.brasoveanu@umft.ro (S.B.); marilena.pirtea@umft.ro (M.P.); ratiu.adrian@umft.ro (A.R.); pirtea.laurentiu@umft.ro (L.P.)

**Keywords:** cesarean scar pregnancy, laparoscopy, temporary clipping of anterior trunk of hypogastric arteries

## Abstract

A cesarean scar ectopic pregnancy (CSP) represents an uncommon yet potentially life-threatening condition requiring immediate and efficient management. We present a case of a 32-year-old woman diagnosed with a scar pregnancy at 8 weeks of gestation. Laparoscopic surgical management was chosen due to its minimally invasive nature and potential for preserving fertility. During the procedure, temporary clipping of uterine arteries was employed to control intraoperative bleeding. The patient recovered well postoperatively with no complications. This case highlights the feasibility and effectiveness of laparoscopic intervention combined with temporary uterine artery clipping in the management of scar pregnancies, offering a valuable approach for clinicians faced with similar cases. Through this report, we aim to contribute to the existing literature on the optimal management of CSP and highlight the efficacy of laparoscopic surgery in this context.

## 1. Introduction

Cesarean scar pregnancy (CSP) embodies a rare yet clinically significant phenomenon, delineated by the implantation of an embryo within the scar tissue ensuing from a previous cesarean section. The predominant clinical manifestation of CSP is vaginal bleeding, which manifests across a spectrum from minimal spotting to substantial hemorrhage. Some women may experience abdominal discomfort or pain, which can be mild to severe. Alterations in menstrual patterns, including irregular periods or persistent spotting, may also manifest. A positive pregnancy test can indicate the presence of the hCG (human chorionic gonadotropin) hormone. There is a worldwide consensus that early diagnosis and appropriate management are essential to prevent complications associated with CSP.

Cesarean scar pregnancy (CSP) represents the rarest form of ectopic pregnancy, with an estimated incidence of 1/1800–1/2500 of all cesarean deliveries performed. However, there are limited data available regarding its occurrence and natural progression. With the rising global rate of cesarean sections, more cases are being diagnosed and reported. Ectopic pregnancy poses a significant threat to life, often resulting in complications such as uterine rupture, severe hemorrhage, hypovolemic shock, and maternal mortality [1]. Early detection and prompt intervention are crucial for a favorable outcome. Transvaginal ultrasound and color flow Doppler imaging offer high diagnostic accuracy. Treatment options vary depending on the individual case and typically involve medical management with methotrexate or surgical intervention [1].

Regarding a recent clinical classification, cesarean scar pregnancy is classified into three types [2]: Type I is characterized by cases where the thickness of the myometrial wall between the sac and the bladder measures more than 3 mm. Type II is described as a thick-walled type, where the thickness of the myometrial wall between the sac and the bladder measures 3 mm or less. Type III refers to a thickness of the myometrium significantly thinner, under 1 mm, or even missing, with a risk of significant bleeding or the potential for placenta previa.

The management of cesarean scar pregnancy encompasses diverse strategies customized to suit individual cases. Timely diagnosis entails expeditious identification through transvaginal ultrasound and color Doppler imaging, facilitating the evaluation of pregnancy location and viability. Potential risks should also be evaluated, including uterine rupture, hemorrhage, and placenta accreta. Many therapeutic options are available, medical and surgical, but the current literature suggests that the laparoscopic approach with ectopic pregnancy resection is a very good option [3].

Medical management involves administration of methotrexate, a medication that halts the growth of the pregnancy, particularly in cases where the condition is detected early, <8 weeks gestation, with human chorionic gonadotropin (hCG) levels < 5000 UI/mL and the patient being hemodynamically stable [4]. Several studies have investigated the efficacy of both local and systemic methotrexate (MTX) for treating cesarean scar pregnancies (CSPs). MTX offers a non-invasive, cost-effective option for patients seeking to preserve fertility. However, it has been linked to a failure rate of 57% and a complication rate of 62.1% [4,5,6].

An amalgamation of uterine artery embolization (UAE) with complementary treatment modalities has demonstrated efficacy in the management of cesarean scar pregnancy (CSP). In a systematic review, UAE combined with dilatation and curettage was highly effective for CSP management, with only 6.4% of cases needing additional treatment and severe complications, such as hemorrhage and hysterectomy, occurring in 3.4% of cases [4,7,8].

Surgical treatment is recommended for patients with signs of hemodynamic instability or failed medical management. Minimally invasive surgical techniques, such as hysteroscopy or laparoscopy, are typically the preferred initial treatment options, although they necessitate surgical skills. One of their advantages is the potential to perform concurrent scar repair during CSP management [3,4].

## 2. Case Report

**Figure 1:**
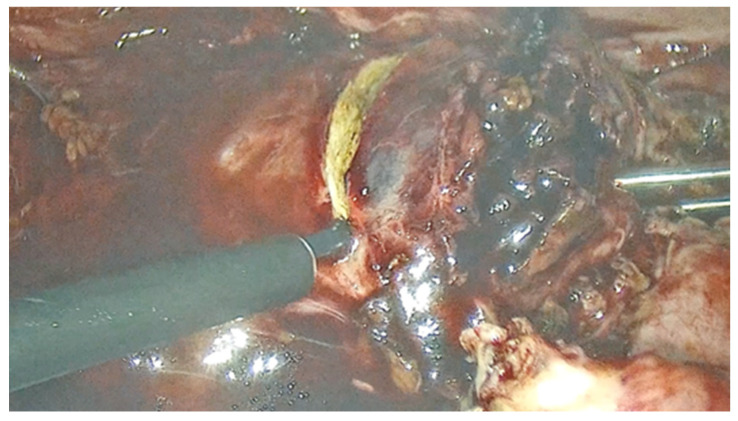
Intraoperative aspect of cesarean scar pregnancy.

**Figure 2:**
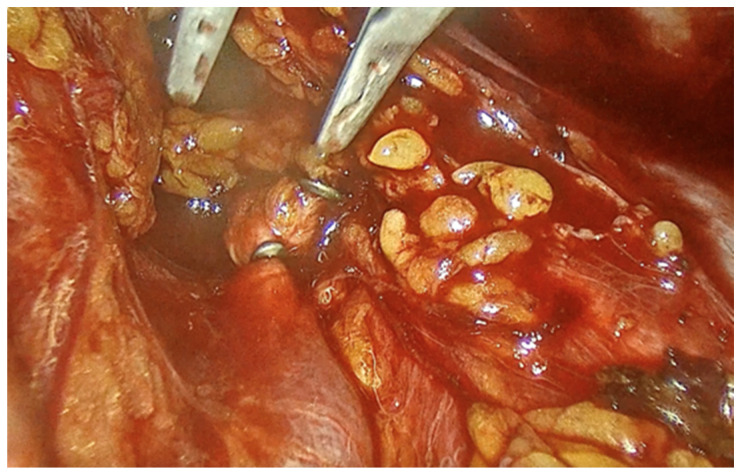
Clipping of the anterior trunk of hypogastric artery.

In this case report, we delineate the clinical presentation of a 32-year-old woman referred to our clinic presenting with 8 weeks of amenorrhea, a positive pregnancy test, and vaginal bleeding. The patient reported a history of cesarean section performed 3 years before. A transvaginal ultrasound was performed. The findings were suggestive for early pregnancy loss. The uterine cavity contained approximately two centimeter of inhomogeneous mass, with only free fluid and clots; no gestational sac or embryo was visualized. After 24 h of persistent bleeding, dilation and curettage were performed. The bleeding stopped and the patient was discharged the following day. After 2 weeks without symptoms, the patient returned with sudden massive bleeding and abdominal pain. Emergency laparoscopic exploration of the peritoneal cavity was performed. During the procedure, we found approximately 200 mL of free blood in the peritoneal cavity and active bleeding from a tumor mass of 3/4 cm located at the level of the left edge of the c-section scar (Figure 1). We decided to remove the mass to repair the scar defect and conserve the uterus. In order to reduce the blood loss, we decided to place temporary clips on the anterior trunk of the internal iliac artery (Figure 2). This procedure was also described in the literature as one that minimizes uterine blood flow during myomectomy [9].

**Figure 3:**
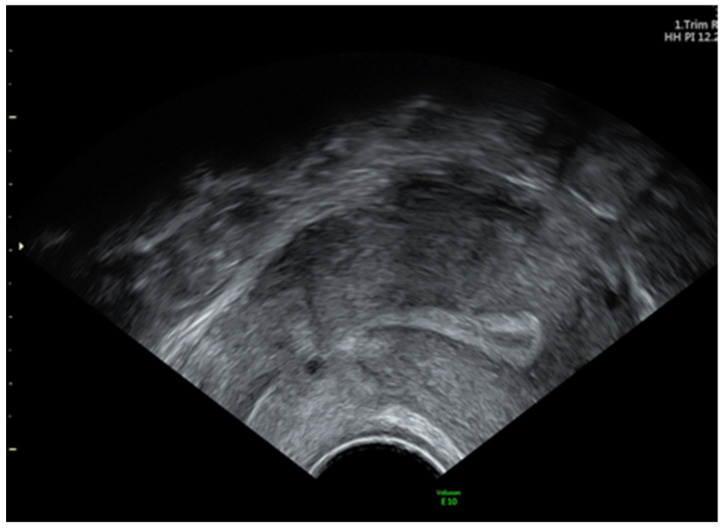
Transvaginal ultrasound image of the uterus in the sagittal plane—6 months after the surgery, demonstrating a completely restored cesarean scar site.

The parietal peritoneum was incised below the lumbo-ovarian ligament. The ureter and anterior trunk of the hypogastric artery were identified in the area where these two structures run parallel. A metallic clip was then placed on the anterior trunk of the hypogastric artery, on each side. The vesico-uterine space was developed, and the tissular mass was removed using a kendo-bag. The margins of the defect were resected in order to obtain viable tissue, and the scar defect was repaired. One single layer of isolated suture was performed using Vycryl 2/0. The recovery was uneventful, and the bleeding stopped after surgery. The patient was discharged after 3 days and followed for 6 months. No more abnormal vaginal bleeding was reported. The ultrasound exam at 6 months revealed good healing of the scar at the level of the anterior uterine wall (Figure 3).

The pathological exam reported the presence of trophoblastic tissue in the mass that was removed from the uterine scar.

## 3. Discussion

The genesis of gynecological laparoscopy is intricately intertwined with the broader historical evolution of laparoscopic surgery. While laparoscopy initially began with diagnostic procedures in various medical fields, its application to gynecology marked a significant advancement in women’s healthcare.

The first recorded gynecological laparoscopy is attributed to the work of German gynecologist Kurt Semm in 1963. Semm pioneered the development of specialized instruments and techniques for laparoscopic gynecological procedures. His innovative contributions laid the groundwork for the widespread adoption of laparoscopy in gynecology [10].

The inaugural instance of laparoscopic intervention for the management of a cesarean scar pregnancy was recorded in 1991. Dr. Jacques Donnez and his team at the Catholic University of Louvain in Belgium successfully treated a patient with a cesarean scar pregnancy using laparoscopic surgery. This landmark case demonstrated the feasibility and effectiveness of laparoscopic intervention in managing this complex condition [11].

These milestones highlight the transformative impact of laparoscopic surgery on gynecological practice, offering less invasive alternatives to traditional open procedures and expanding treatment options for a range of gynecological conditions.

Since its initial report in 1978 [12], cesarean scar pregnancy (CSP) has been recognized as a long-term risk associated with cesarean delivery. CSP constitutes approximately 6% of all ectopic pregnancies, with an estimated incidence ranging from 1:1800 to 1:2500 in women who have undergone cesarean deliveries [13]. Inadequately managed CSP can result in severe complications, predominantly hemorrhagic, posing a threat to the woman’s life. Various therapeutic approaches exist; local excision appears to be the most effective option. In the hands of experts, the laparoscopic approach is arguably the optimal surgical choice, allowing for precise tissue dissection, electrosurgical hemostasis, and vascular control with minimal invasiveness. Given the risk of severe intraoperative bleeding, retroperitoneal vascular control is essential during this surgery [13].

Due to its infrequency, precise diagnosis and prompt intervention are imperative to avert potential serious complications in cases of cesarean scar pregnancy (CSP). Previous studies discourage expectant management due to its low success rate and unfavorable prognosis. Termination of CSP in the first trimester following diagnosis is typically recommended, particularly for hemodynamically stable cases, which may benefit from more conservative management approaches [14].

Most cesarean scar ectopic pregnancies are asymptomatic, although a subset may manifest mild symptoms such as light vaginal bleeding or abdominal discomfort. Diagnosis often relies on ultrasound criteria, particularly transvaginal ultrasound supplemented with transabdominal ultrasound. Management evidence predominantly stems from case reports and small series, advocating any method that removes pregnancy and scar tissues to improve outcomes. Both medical (methotrexate injection) and surgical approaches are used, with a focus on early termination and multidisciplinary care [15].

Currently, there is no universally optimal approach for managing cesarean scar pregnancy (CSP) worldwide. However, medical management, primarily through methotrexate (MTX), has shown superior outcomes compared to the expected treatment. Local MTX injection guided by transvaginal sonography is recommended as the initial therapy for CSP patients with human chorionic gonadotropin (hCG) levels below 100,000 IU/L [16]. Asymptomatic, hemodynamically stable, and unruptured pregnancies at less than eight weeks of gestational age are suitable candidates for methotrexate treatment [15]. This approach boasts a relatively high success rate (73.9% for single injections and 88.5% for multiple injections) and a low complication rate (approximately 9.6%) [16,17,18].

A systematic review and meta-analysis were undertaken to assess the efficacy and safety of uterine artery embolization followed by curettage in contrast to methotrexate plus curettage for the treatment of cesarean scar pregnancy in China. The combination of UAE and curettage significantly decreased the time required for β-hCG normalization and hospital stay, as well as reduced blood loss and adverse events compared to MTX plus curettage [19]. Although, previous studies have reported an adverse effect on endometrial and ovarian function of UAE [16,20].

In a recent national cohort study conducted in the United Kingdom, surgery has been associated with a notable success rate (96%), a low incidence of complications (36%), and abbreviated post-treatment follow-up periods [21].

Laparoscopic surgery confers numerous advantages in the management of cesarean scar pregnancy (CSP), encompassing precise visualization, minimal invasiveness, and favorable cosmetic outcomes. This case underscores the efficacy and safety of laparoscopic surgical management in carefully selected patients with CSP. In our case, the patient underwent a successful laparoscopic excision of the tissue retained, and uterine defect was repaired. She had an uneventful recovery and was able to maintain her future fertility. The particularity of this case is represented by the fact that the diagnosis of an ectopic scar pregnancy was established only after the surgery, and the initial assumption was incomplete abortion. This case can be a warning sign that, in patients with previous c-section, the possibility of having an ectopic scar pregnancy should be taken into consideration as a possible differential diagnosis.

Moreira et al. present a similar case of an incomplete abortion at 18 weeks gestation. After dilatation and evacuation, the patient presented with severe abdominal pain. A laparoscopic approach with temporary internal iliac artery ligation was performed to treat the very large volumes of tissue sequestered by an isthmocele. Blood loss was minimal, and the patient was discharged the same day [22].

Rudaitis presents a case involving a woman diagnosed with a complicated cesarean scar pregnancy, meeting the criteria for the type 3 classification of CSP. Initially, expectant management was chosen; however, due to complications arising from the CSP, secondary surgical intervention via laparotomy became necessary [23].

In a retrospective study conducted by Lin et al., it was concluded that the laparoscopic approach is deemed appropriate for all cases classified as a type 3 cesarean scar pregnancy (CSP), yielding favorable outcomes [16]. The findings were consistent with previous studies, which indicated that the laparoscopic approach was linked to a higher success rate [2,16,24].

In addition, numerous case reports detailing patients diagnosed with CSP, even in the absence of bleeding, lend support to our surgical management approach [25]. Surgery is advantageous in minimizing recurrence by removing the previous scar tissue and ensuring the repair of the scar defect, a very important aspect for patients that desire future pregnancies. Scar pregnancy presents a unique clinical challenge due to the potential for severe hemorrhage and uterine rupture. Laparoscopic surgery offers a safe and effective approach for scar pregnancy management, providing excellent visualization and precise tissue dissection while minimizing surgical trauma.

## 4. Conclusions

Laparoscopic surgical management emerges as a viable and effective option for the treatment of cesarean scar ectopic pregnancy. Early diagnosis and individualized treatment strategies are imperative for optimizing outcomes in patients with CSP. This case report contributes valuable insights to the existing literature on CSP management, emphasizing the pivotal role of laparoscopic surgery in achieving favorable clinical outcomes.

## Data Availability

Data are contained within the article.
